# Comparison of Effects of Metformin, Phenformin, and Inhibitors of Mitochondrial Complex I on Mitochondrial Permeability Transition and Ischemic Brain Injury

**DOI:** 10.3390/biom10101400

**Published:** 2020-10-01

**Authors:** Kristina Skemiene, Evelina Rekuviene, Aiste Jekabsone, Paulius Cizas, Ramune Morkuniene, Vilmante Borutaite

**Affiliations:** Neuroscience Institute, Lithuanian University of Health Sciences, LT-50161 Kaunas, Lithuania; kristina.skemiene@lsmuni.lt (K.S.); evelina.rekuviene@lsmuni.lt (E.R.); aiste.jekabsone@lsmuni.lt (A.J.); paulius.cizas@lsmuni.lt (P.C.); ramune.morkuniene@lsmuni.lt (R.M.)

**Keywords:** metformin, phenformin, brain ischemia, hypoxia, mitochondrial complex I, permeability transition

## Abstract

Damage to cerebral mitochondria, particularly opening of mitochondrial permeability transition pore (MPTP), is a key mechanism of ischemic brain injury, therefore, modulation of MPTP may be a potential target for a neuroprotective strategy in ischemic brain pathologies. The aim of this study was to investigate whether biguanides—metformin and phenformin as well as other inhibitors of Complex I of the mitochondrial electron transfer system may protect against ischemia-induced cell death in brain slice cultures by suppressing MPTP, and whether the effects of these inhibitors depend on the age of animals. Experiments were performed on brain slice cultures prepared from 5–7-day (premature) and 2–3-month old (adult) rat brains. In premature brain slice cultures, simulated ischemia (hypoxia plus deoxyglucose) induced necrosis whereas in adult rat brain slice cultures necrosis was induced by hypoxia alone and was suppressed by deoxyglucose. Phenformin prevented necrosis induced by simulated ischemia in premature and hypoxia-induced—in adult brain slices, whereas metformin was protective in adult brain slices cultures. In premature brain slices, necrosis was also prevented by Complex I inhibitors rotenone and amobarbital and by MPTP inhibitor cyclosporine A. The latter two inhibitors were protective in adult brain slices as well. Short-term exposure of cultured neurons to phenformin, metformin and rotenone prevented ionomycin-induced MPTP opening in intact cells. The data suggest that, depending on the age, phenformin and metformin may protect the brain against ischemic damage possibly by suppressing MPTP via inhibition of mitochondrial Complex I.

## 1. Introduction

Ischemia-induced brain injury is serious, life-threatening pathology which may cause death or severe complications such as physical disability and cognitive impairment. The incidence of stroke is increasing and seeks about 15 million new strokes worldwide each year [1]. Even when patients survive acute stroke episodes, neurological consequences may cause disability which negatively affect the quality of patients life, and that becomes an important issue as well. On the other hand, hypoxia at birth may affect developing brains and this can also have a devastating effect on the growth and maturation of the central nervous system. Therefore, it is important to explore the possibilities not only to reduce stroke mortality, but also to ease the consequences of hypoxic/ischemic episodes for the patients’ health and disability. Consequently, new therapies are constantly being sought and one of the research directions is a search for effective neuroprotective drugs. For this purpose, understanding of the molecular and cellular mechanisms of ischemia-induced injuries is crucial.

Mitochondrial injury and opening of the mitochondrial permeability transition pore (MPTP) has been suggested as a key mechanism of irreversible damage during ischemia (for review see [2,3,4,5]. Oxygen and substrate deficiency acutely impairs oxidative phosphorylation, while prolonged ischemia results in irreversible uncoupling of oxidative phosphorylation, energy depletion, opening of MPTP and necrotic cell death [6,7]. In several studies a decade ago, it has been observed that reversible inhibition of Complex I of mitochondrial electron transfer system may exert cardioprotective effect against ischemic damage possibly via suppression of reverse electron transfer and inhibition of production of reactive oxygen species (ROS) [8,9]. There were also studies suggesting that Complex I activity may modulate MPTP opening [10]. Though the structure and regulation of MPTP is still the hot issue of the research [11,12,13], it has been demonstrated that MPTP suppression by cyclosporine A and inhibitors of mitochondrial Complex I decrease cell death in cardiomyocytes, U937 and KB cells [14,15]. Opposing data showed different sensitivity of MPTP to cyclosporine A and inhibitors of Complex I in various tissues [16]. It is still unclear whether MPTP in the brain can be modulated by the activity of Complex I and whether this exerts any neuroprotective effect during ischemia/reperfusion.

Metformin is one of the well-known biguanide derivatives and is widely used as anti-diabetic drug decreasing hepatic glucose output by inhibiting gluconeogenesis [17]. Depletion of energy reserves in the liver activates AMP-dependent protein kinase (AMPK) [18,19] and inhibits adenylate cyclase [20], resulting in a decrease in blood glucose and an increased sensitivity to insulin. Besides this action, metformin has been suggested to activate AMPK signaling pathway [10] via mild inhibition of mitochondrial Complex I and to reduce tumorigenesis due to effects on AMPK activity and reactive oxygen species (ROS) formation [21]. It has also received attention as a possible neuroprotective agent. In clinical studies on patients with diabetes, the treatment with metformin has been shown to reduce the risk of stroke [22]. Also, there are data that therapy of metformin applied to diabetic patients improved prognosis and severity of neurological damages of acute ischemic stroke [23]. The molecular mechanisms of metformin action during stroke are unclear but may include inhibition of endogenous ROS production and oxidative stress [24,25], up-regulation of AMPK and down-regulation of expression of apoptotic proteins such as Bax and caspase 3 [26]. On the other hand, recent studies suggest that metformin can exert protective effects against cardiac ischemia/reperfusion-induced damages independently of AMPK activation but inhibiting Complex I and MPTP [10]. Whether similar mechanisms may operate in the brain and exert neuroprotective effects during brain ischemia is not investigated.

Another derivative of biguanides, phenformin, is no longer used as anti-diabetic drug in clinical practice due to its toxicity during long-term treatment. It has been demonstrated that phenformin has a stronger inhibitory effect on mitochondrial Complex I than metformin possibly due to its hydrophobic properties and easier transport into mitochondria [19]. Phenformin has been also shown to inhibit complexes II and IV of the mitochondrial electron transfer system [27].

There is some evidence suggesting that sensitivity to ischemic damage may be different in developing and aging individuals. Studies with aged animals showed reduced cardiac mitochondrial Ca^2+^ handling [28], increased sensitivity to calcium overload [29] and increased susceptibility to Ca^2+^-induced MPTP opening, associated with an elevated release of cytochrome c and other mitochondrial proteins, compared with young animals [30,31]. An enhanced susceptibility to calcium-induced MPTP opening and cell death was also shown in aging rodent brains [32,33,34], and this may change the response of cerebral cells to hypoxia/ischemia. Consequently, effects of mitochondria-targeted compounds may also vary with the age of individuals.

In this study, we sought to get deeper insight into the mechanism of neuroprotective action of biguanide derivatives and aimed to investigate whether metformin, phenformin, and inhibitors of mitochondrial Complex I can prevent cell death by inhibiting opening of MPTP in ischemia-affected brain slice cultures from premature and adult rats.

## 2. Materials and Methods

### 2.1. Preparation and Cultivation of Organotypic Brain Slices

All experimental procedures were reviewed and approved by the National Ethical Committee for Animal Care (Licenses No 0217 and No 0228) according to Directive 2010/63/EU of the European Parliament. The rats were maintained and handled at Lithuanian University of Health Sciences animal house in agreement with the Guide for the Care and Use of Laboratory Rats. Brain slices were prepared from the cerebral hemispheres of 5–7-days and 2–3-months old Wistar rats. The animals were anesthetized with inhalation of CO_2_, after cervical dislocation sculls were dissected, brains removed and placed in 4 °C PBS with 10 mM glucose. The cerebral hemispheres were separated from cerebella and meninges were removed. The brains were immobilized caudal end down and sectioned with the vibratome (Vibratome 1000, Technical Products International Inc., Saint Louis, MO, United States) in PBS and glucose-filled cooling bath at 1 °C. The brains were coronally sliced with low profile microtome blade (Leica Biosystems) into 300 µm thick slices with an amplitude of 2 mm and minimal advancing speed. The slices selected for culturing were collected from vibratome bath and placed on porous tissue culture inserts (BD-Falcon, 1 µm pores) in 6-well insert-adapted cell culture plates (BD-Falcon) at the air–liquid interface with medium. Medium for cultivating organotypic brain slices from 5–7 days old rats was Dubelco’s Modified Eagle Medium (DMEM) with Glutamax (Gibco) supplemented with 12% horse serum, 12% fetal bovine serum, Antibiotic-Antimycotic (10 units/mL-10 µg/mL) and HEPES (25 mM). Medium for cultivating organotypic brain slices from 2–3 months old rats was DMEM with Glutamax (Gibco) supplemented with 2.5% horse serum, 2.5% fetal bovine serum, Antibiotic-Antimycotic (10 units/mL-10 µg/mL) and HEPES (25 mM). The slices from 5–7 days old rats were cultivated for 1 week and slices from 2–3 months old rats for 3 days in the incubator (37 °C, humidified atmosphere, 5% CO_2_/95% air) before the start of treatments.

### 2.2. Preparation of Cultures of Cerebellar Granule Cells

Cultures of cerebellar granule cells (CGC) were prepared from 7 to 8 days old Wistar rats as described in [35]. Cells were grown in vitro for 6–7 days at density 0.25 × 10^6^ cells/cm^2^ in 24-well plates in cell culture growth medium (DMEM Glutamax supplemented with 5% fetal bovine serum, 5% horse serum, 13 mM glucose, 20 mM KCl, and 1% penicillin/streptomycin) coated with 0.001% poly-l-Lysine, (Sigma-Aldrich). Cells were maintained at 37 °C in a humidified atmosphere containing of 5% CO2/95% air. To inhibit proliferation of glial cells CGC cultures were treated with 10 µM of cytosine β- D–arabinofuranoside (Ara-C) at 1 DIV. These cultures (called pure neuronal cultures) contained 97.2 ± 0.4% neurons, 0.3 ± 0.1% microglia, and 2.4 ± 0.4% astrocytes.

### 2.3. Simulated Ischaemia Model

After one week (slice cultures from 5–7 days old rats) or after 3 days (slice cultures from 2–3 months old rats) ex vivo brain slice cultures were subjected to 24 h simulated ischemia by placing them in a humidified chamber perfused by gas mixture of 2% O_2_/5% CO_2_/93% N_2_ in the presence/absence of 10 mM D-deoxyglucose (DOG).

### 2.4. Determination of Lactate Dehydrogenase Activity in the Incubation Medium

For the evaluation of necrosis, activity of lactate dehydrogenase (LDH) released into the brain incubation medium was measured spectrophotometrically recording NADH oxidation rate at 340 nm wavelength. The incubation medium of the slices (control group and ischemic groups) were collected and centrifuged at 10,000× *g* for 20 min. Aliquots of the medium were added to the buffer containing 0.1 M TRIS-HCl (pH 7.5), 1 mM potassium salt of pyruvic acid, 0.1 mM NADH. LDH release was calculated from the rate of the decrease of absorbance and expressed as nmol NADH/min/mg brain slice tissue [36].

### 2.5. Evaluation of Cell Viability by Fluorescence Microscopy

The viability of cells in organotypic brain slice cultures was assessed by propidium iodide (PI, 7 µM) staining using an inverted fluorescence microscope OLYMPUS IX71S1F-3 (Olympus Corporation, Tokyo, Japan), x20 objective. Pictures of at least 5 randomly selected fields per slice (2 slices per group of individual experiment) were taken. Pictures were analyzed by ImageJ freeware, version 1.49, and raw data expressed as the mean level of red fluorescence intensity.

Neuronal viability in CGC cultures was assessed by staining with Hoechst-33342 (4 µg/mL) and PI (7 µM). Neurons were recognized according to characteristic shape and morphology by phase contrast microscopy. Cells with homogeneously Hoechst-33342 stained nuclei were classified as viable, with condensed/fragmented chromatin as apoptotic and PI-positive cells as necrotic. Cells were counted in at least 5 randomly selected microscopic fields in each well, 2 wells per treatment.

### 2.6. Isolation of Brain Mitochondria

Brain mitochondria were isolated by differential centrifugation [37]. The brains were cut into small pieces and homogenized with a Teflon-glass homogenizer in the isolation buffer containing 222 mM manitol, 75 mM sucrose, 5 mM HEPES, 1mM EGTA (pH 7.4, 4 °C). Cytosolic and mitochondrial fractions were separated by differential centrifugation (5 min at 1000× *g*, then 10 min at 10,000× *g*). Total cytosolic and mitochondrial protein was measured by the Biuret method.

### 2.7. Measurement of Mitochondrial Respiration

Mitochondrial respiration was measured with high-resolution respirometry OROBOROS Oxygraph-2k (Oroboros Instruments, Insbruck, Austria) at 37 °C in 2 mL respiration buffer containing 110 mM KCl, 10 mM Tris-HCl, 5 mM KH_2_PO_4_, 2.24 mM MgCl_2_, pH 7.2 and 0.25 mg/mL mitochondrial protein. Respiration assay started with addition of pyruvate/malate (1/1 mM) and mitochondria (0.25 mg/mL), this respiration represented proton leak-driven respiration (LEAK) [38]. Then the respiration buffer was supplemented with 2 mM ADP and ADP-stimulated phosphorylating respiration (OXPHOS) was registered with pyruvate/malate. Rates of oxygen consumption were expressed in pmol O_2_/s/mg mitochondrial protein.

### 2.8. Measurement of Mitochondrial Calcium Retention Capacity

Calcium retention capacity (CRC) of brain mitochondria was determined fluorometrically (Fluorescence Spectrometer Perkin Elmer LS55) using 100 nM Calcium Green 5N (excitation at 507 nm, emission at 536 nm) in medium containing 200 mM sucrose, 10 mM Tris-HCl, 1 mM KH_2_PO_4_, 10 μM EGTA and 5 mM succinate, pH 7.4 at 37 °C as described in [39]. Isolated mitochondria (0.2 mg protein/mL) were incubated with rotenone (0.05 and 1 µM), amobarbital (2.5 mM), cyclosporine A (0.5 µM) for 2 min or phenformin (Phen, 0.1 and 1 mM), metformin (1 and 10 mM) for 5 min and then pulses of 1.67 µM CaCl_2_ were applied in 2 min intervals. Calcium ions were continually loaded until large increase of Calcium Green 5N fluorescence was observed which indicated the release of stored in mitochondria calcium due to opening of MPTP.

### 2.9. Assesment of MPTP Opening in Neuronal Cell Cultures.

For the experiments, pure neuronal cell cultures (see Section 2.2) were pre-incubated 1 h with 50–100 µM phenformin, 2–3 mM metformin, 0.05–1 µM rotenone, or 3–5 μM cyclosporine A. After incubation, cells were washed and resuspended in modified Hank’s Balanced Salt Solution (HBSS) containing 10 mM HEPES, 92 mM L-glutamine and 100 µM succinate. Cells were loaded with 1 µM Calcein-AM (Invitrogen) for 30 min at 37 °C in the dark. Then 1 mM CoCl_2_ (ChemCruz) was added and cells were incubated for another 15 min. Then cells were twice washed in warm HBSS buffer to remove residual dye and imaged with a fluorescence microscope. To induce MPTP opening, 1 μM ionomycin was added and changes in fluorescence were measured at 5 min intervals. Cells were imaged in at least five microscopic fields per well (2 wells per condition). Image analyses were performed using ImageJ software and results were expressed as fluorescence arbitrary units per cell.

### 2.10. Measurement of the Mitochondrial Electron Transfer System Complex I Activity

The activity of mitochondrial electron transfer system Complex I was determined in brain mitochondria isolated from 5–7 days and 2–3 months aged rats spectrophotometrically at a wavelength of 340 nm following the oxidation of 100 µM NADH in the presence of 2 µg/mL antimycin, 2 mM sodium azide, 3 mg/mL bovine serum albumin (BSA), 60 µM coenzyme Q1 (CoQ1) and investigated biguanides—phenformin or metformin—in a medium containing 25 mM KH_2_PO_4_ (pH 7.4 at 37 °C) and 0.125 mg/mL freeze–thawed mitochondria. Complex I activity was calculated by substracting nonspecific NADH oxidation rate (obtained after rotenone supplement) from total NADH oxidation rate and expressed as nmol NADH/min/mg protein [40].

### 2.11. Statistical Analysis

All data are expressed as means ± standard errors of at least three experiments on separate preparations of brain slice or CGC cultures. The graphs were created and statistical significance was evaluated by SigmaPlot v13 software. Data were tested for normality using Shapiro–Wilk test and statistically compared between experimental groups by One Way Anova. Data presented in Figure 2a and Figure 7 did not pass Shapiro–Wilk normality test, and were tested by One Way Anova using Fisher LSD test. A value of *p* < 0.05 was considered statistically significant.

## 3. Results

### 3.1. Effect of Phenformin and Metformin on Neuronal Viability in CGC Cultures

**Figure 1:**
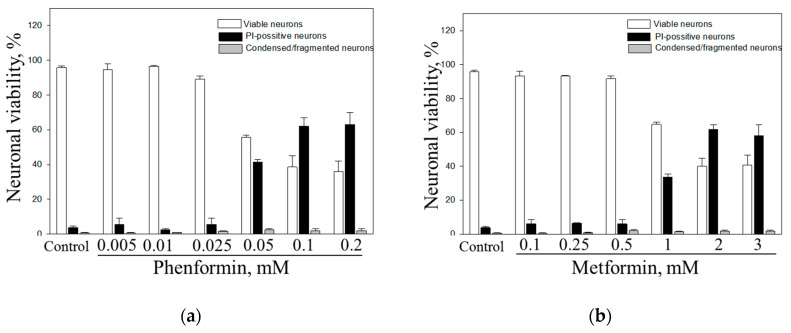
Effect of phenformin (**a**) and metformin (**b**) on neuronal viability in cerebellar granule cells (CGC) cultures. CGC were treated with phenformin and metformin at indicated concentrations. White column—viable neurons (%), black—necrosis (%), grey—apoptosis (%). The number of all neuronal cells (live, apoptotic and necrotic) in the vision field was equal to 100%. The data are presented as means ± standard errors of 3 experiments on separate CGC cultures.

Phenformin and metformin are known to inhibit mitochondrial Complex I, which if complete and prolonged may lead to neuronal death. To find the optimal concentrations that are not toxic to neurons over 24 h incubation, we investigated effects of phenformin (0.005–0.2 mM) and metformin (0.1–3 mM) on viability of neurons in CGC cultures. The numbers of viable, apoptotic and necrotic cells were counted and expressed as percentage of all neurons counted (see Figure 1 in result section). As shown in (Figure 1A) phenformin at 0.05–0.25 mM concentrations had no effect on neuronal viability: There was only negligible percentage of necrotic cells in the cultures −2.5–9.5%, which was not different from percentage in the control cultures −3.7%. Higher concentrations of phenformin (0.5, 1 and 2 mM) caused an increase in neuronal necrosis in CGC cultures compared to the control group by 41%, 62%, and 63%, respectively. At any concentration, phenformin did not induce neuronal apoptosis in CGC cultures.

The data presented in Figure 1b show that metformin concentrations up to 0.5 mM have no effect on neuronal viability in CGC cultures: The level of necrosis at these concentrations did not exceed 6.6%. At higher concentrations of metformin, 1, 2, and 3 mM, an increase of necrotic neuronal death was observed by 33%, 61% and 58%, respectively (Figure 1b). Metformin did not cause neuronal apoptosis at any concentration investigated.

Based on these findings 0.025 mM phenformin and 0.5 mM metformin were chosen as the highest non-toxic concentrations to be used in further experiments on brain slice cultures.

### 3.2. Effects of Phenformin and Metformin on Simulated Ischemia- and Hypoxia-Induced Necrosis in Brain Slice Cultures

**Figure 2:**
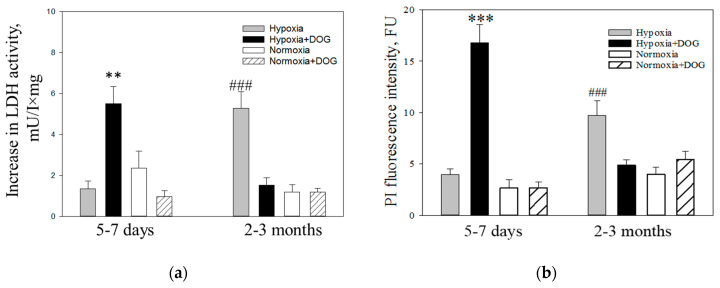

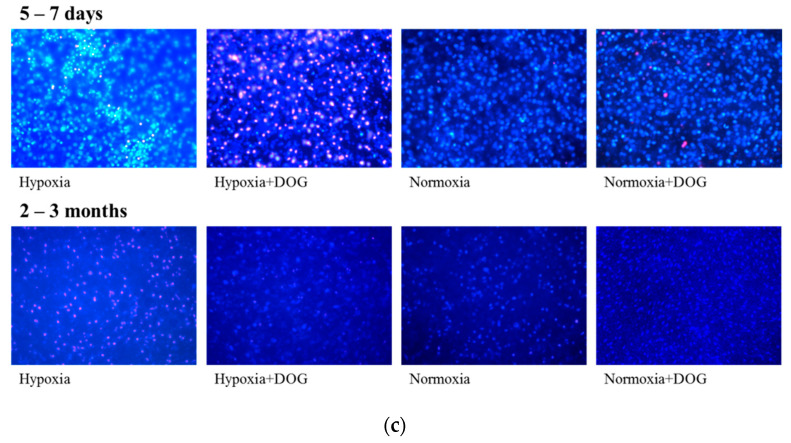
The effect of hypoxia and simulated ischemia on cell death in brain slice cultures from 5–7 days and 2–3 months old rats. After 24 h hypoxia or hypoxia with 10 mM D-deoxyglucose (DOG), the level of necrosis was evaluated by measuring increase in lactate dehydrogenase (LDH) activity in the slice culture medium (**a**) and PI fluorescence in the slice cultures (**b**). FU—arbitrary fluorescence units. Representative fluorescence microscopy images of brain slice cultures stained with PI (red fluorescence) and Hoechst-33342 (blue fluorescence) are presented in (**c**). The data are presented as means ± standard errors of 7–18 experiments on separate brain slices cultures. ∗∗—*p* < 0.01; ∗∗∗—*p* < 0.001 compared to normoxic control with DOG, ###—*p* < 0.001 compared to normoxic control without DOG.

Brain slice cultures were used in further experiments as they are considered to preserve complexity of brain tissue architecture and maintain native synaptic circuitry which is important investigating effects on pharmacological compounds [41]. First, we investigated the effects of 24 h hypoxia and 24 h simulated ischemia (hypoxia plus DOG to inhibit glycolysis) on cell viability in brain slice cultures prepared from premature (5–7 days old) and adult (2–3 months old) rats. The level of necrosis in these experiments was determined by measuring LDH release into the incubation media of the slice cultures. As can be seen in Figure 2a, simulated ischemia (indicated as hypoxia with DOG) caused about 5-fold increase in LDH release into 5–7 days rat brain slice culture media compared to the normoxia with DOG group (5.5 ± 0.8 and 0.9 ± 1.1 mU/L×mg, respectively) (Figure 2a). Twenty-four hour hypoxia without DOG had no effect on LDH release in brain slices cultures of 5–7 days old rats compared with the normoxia group (1.4 ± 0.3 and 2.3 ± 2.2 mU/L×mg, respectively). Similar results were obtained measuring necrosis according to PI staining of cell nuclei: 24 h hypoxia with DOG caused 5-fold increase of PI fluorescence intensity in 5–7 days old rat brain slice cultures compared to the normoxia with DOG group (16.8 ± 1.8 and 2.6 ± 0.6 FU, respectively) (Figure 2b). Twenty-four hour hypoxia without DOG had no effect on brain slices cultures from premature rats compared with the normoxic group (3.9 ± 0.6 and 2.6 ± 0.8 FU, respectively).

Unlike 5–7 days age group, the level of LDH in media from 2–3 months old rat brain slice cultures after simulated ischemia was not statistically significantly different from the corresponding control group (1.4 ± 0.2 and 1.7 ± 0.3 mU/L×mg, respectively). Meanwhile, 24 h hypoxia without DOG caused 2-fold increase in LDH release as compared to control group (3.9 ± 0.2 and 1.7 ± 0.19 mU/L×mg, respectively) (Figure 2a), suggesting that adult brain slices cultures exhibit extreme sensitivity to hypoxia alone. Interestingly, hypoxia-induced brain damage at this age was prevented by DOG treatment (Figure 2a). Measurement of PI fluorescence intensity in brain slice cultures of 2–3 months rats revealed that there was a similar increase in hypoxia-induced necrosis compared with normoxia as it was observed in the measurements of LDH release (Figure 2a,b): In slices exposed to hypoxia alone the level of fluorescence was increased to 9.7 ± 1.4 FU compared to 3.9 ± 0.6 FU in normoxia, whereas fluorescence in slices exposed to hypoxia in the presence of DOG was 4.8 ± 0.5 FU which is at the same level as in normoxia plus DOG −4.0 ± 0.7 FU (Figure 2b,c). These data suggest that slice cultures from adult brains are more sensitive to hypoxia alone than premature brains and that hypoxia-induced necrosis in adult brains can be prevented by DOG. As both methods—determination of necrosis by LDH release and PI fluorescence, gave the same results, in further experiments we evaluated necrosis according to LDH release into slice culture media.

**Figure 3:**
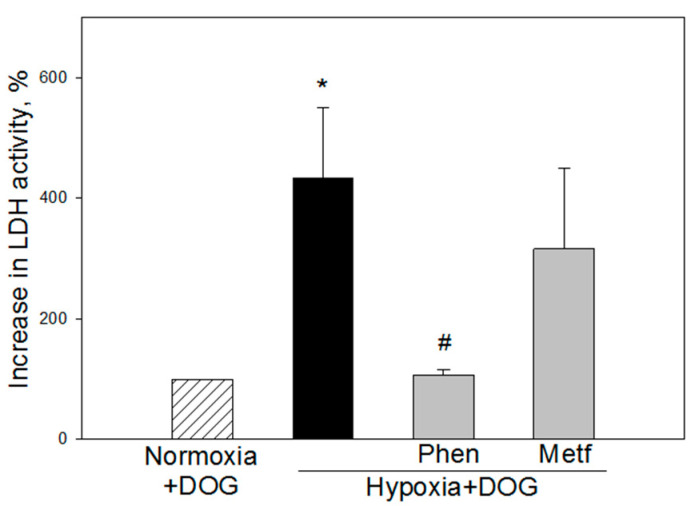
Effect of phenformin and metformin on simulated ischemia-induced cell death in brain slice cultures from 5–7 days old rats. Where indicated, 0.025 mM phenformin (Phen) or 0.5 mM metformin (Metf) was added to slice culture incubation medium before the start of simulated ischemia as described in Methods. The level of necrosis after simulated ischemia, indicated as treatment with 10 mM DOG and 24 h hypoxia, was evaluated by measuring increase in LDH activity in slice culture medium. The data were normalized against normoxic controls of each group assuming that normoxic level is 100% and presented as means ± standard errors of 8 separate experiments. *—*p* < 0.05 compared with normoxic control with DOG, #—*p* < 0.05 compared to hypoxia with DOG.

When 5–7 days brain slices cultures were preincubated with 0.025 mM phenformin and then exposed to 24 h simulated ischemia (hypoxia plus DOG), the level of necrosis was substantially decreased, as measured by LDH release (106 ± 10%) compared to hypoxia+DOG group (434 ± 115%) (Figure 3). Preincubation of slices with 0.5 mM metformin had no statistically significant effect and the level of LDH release remained similar as in the simulated ischemia group (316 ± 134%) (Figure 3).

**Figure 4:**
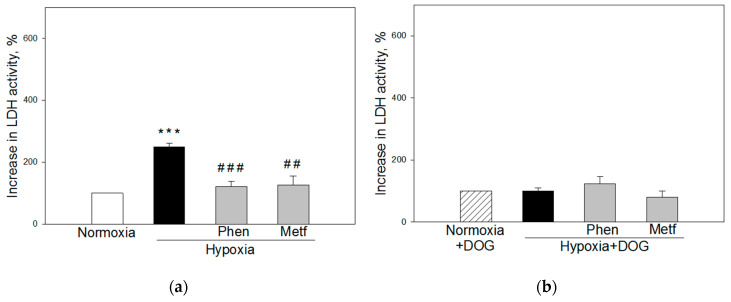
Effect of phenformin and metformin on hypoxia-induced (**a**) and simulated ischemia-induced (**b**) necrosis in brain slice cultures from 2–3 months old rats. Where indicated, 0.025 mM of phenformin (Phen) or 0.5 mM of metformin (Metf) was added to slice culture incubation medium before the start of hypoxia (**a**) or simulated ischemia (**b**) as described in Methods. The level of necrosis after hypoxia or simulated-ischemia was evaluated by measuring increase in LDH activity in slice culture medium. The data were normalized against normoxic controls of each group assuming that normoxic level is 100% and presented as means ± standard errors of 7–9 separate experiments. ***—*p* < 0.001 compared normoxic control without DOG, ##—*p* < 0.01; ###—*p* < 0.001 compared to hypoxia without DOG.

In 2–3 month old rats brain slice cultures, preincubation with 0.025 mM phenformin and 0.5 mM metformin before hypoxia caused 2-fold decrease in LDH release (121 ± 16 and 126 ± 29%, respectively) compared with the hypoxia group (250 ± 12%) (Figure 4a). Under simulated ischemia conditions, metformin and phenformin had no effect on LDH release which was at the same level as at normoxia and hypoxia plus DOG (Figure 4b).

Altogether, the data suggest that metformin and phenformin reduce hypoxia-induced necrotic cell death in brain slice cultures of adult rats, while in brain slices of premature animals only phenformin has the protective effect.

**Figure 5:**
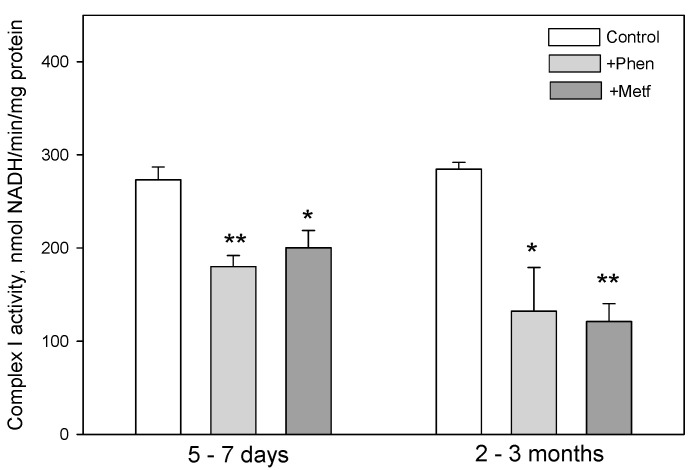
Effect of phenformin and metformin on Complex I activity of brain mitochondria isolated from 5–7 days and 2–3 months age rats. Complex I activity was evaluated by following NADH oxidation rate in isolated freeze–thawed brain mitochondria without (control) or with biguanides—phenformin (Phen, 0.025 mM) or metformin (Metf, 0.5 mM) as described in Methods. Statistically significant difference compared with control group: *—*p* ≤ 0.05, **—*p* ≤ 0.01 (n = 3).

To test whether biguanides may act via inhibition of Complex I, we evaluated the effect of phenformin and metformin on the activity of Complex I in isolated brain mitochondria of both age groups. Data presented in Figure 5 show that 0.025 mM phenformin and 0.5 mM metformin directly suppress the rate of NADH oxidation by Complex I of premature (by 34% and by 27%, respectively) and adult (by 54% and by 58%, respectively) rat brain mitochondria compared with the respective control group. Using another model of global brain ischemia *in vitro,* we have found that mitochondrial Complex I activity was inhibited by 49% in prematures and by 40% in adult brains after 2 h ischemia, and that direct addition of phenformin and metformin to isolated ischemia-affected mitochondria did not cause additional inhibitory effect on Complex I (unpublished data; data not shown).

### 3.3. Modulation of Simulated Ischaemia-Induced Necrosis by Inhibitors of Complex I and MPTP in Brain Slice Cultures

**Figure 6:**
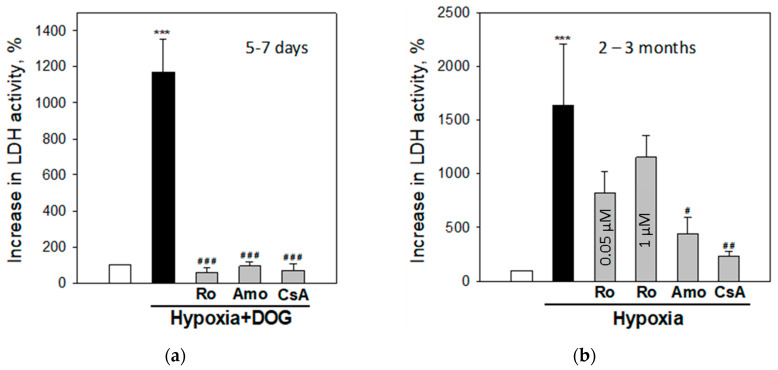
The effect of mitochondrial respiratory Complex I and mitochondrial permeability transition pore (MPTP) inhibitors on the level of simulated ischemia-induced necrosis in brain slice cultures from 5–7 days (**a**) and 2–3 months old (**b**) rats. Where indicated, 0.05 μM or 1 μM rotenone (Ro), and 2.5 mM amobarbital (Amo), and 0.5 mM cyclosporin A (CsA) were added to slice culture incubation medium before the start of simulated ischemia as described in Methods. The level of necrosis in slice cultures was evaluated by measuring increase in LDH activity in slice culture medium. The data were normalized against normoxic controls of each age group assuming that normoxic level is 100% (white bars) and presented as mean+standard error. N = 3–7; ***—statistically significant difference compared with normoxic control of the corresponding group, *p* ≤ 0.001. ###, ## and #—statistical significance compared to hypoxia +DOG or hypoxia treatment, *p* ≤ 0.001, *p* ≤ 0.01 and 0.05, respectively.

To test the involvement of mitochondrial respiratory Complex I and MPTP in simulated ischemia-induced damage in brains from different age groups, classical Complex I inhibitors rotenone (0.05 µM) and amobarbital (2.5 mM), and an inhibitor of MPTP 0.5 µM CsA were added to brain slice incubation media before simulated ischemia. As can be seen in Figure 6a, rotenone, amobarbital, and CsA completely prevented simulated ischemia-induced LDH release in brain slices from 5–7 days: The LDH release to the medium was the same as in the normoxic control (Figure 6a).

The effects of Complex I and MPTP inhibitors were also tested on hypoxia-induced LDH release in the samples from 2–3 months old animals. As presented above, hypoxia alone resulted in pronounced necrosis which was fully blocked by DOG. Rotenone used at 0.05 µM (the concentration effective in other age group during simulated ischemia) and at 1 µM, did not prevent hypoxia-induced necrosis measured as LDH release, though some tendency of protection was seen at lower rotenone concentration (Figure 6b). Also, 1µM rotenone itself was slightly toxic to normoxic samples of 2–3 months old rats inducing 1.5-fold increase in LDH release compared to control normoxic cultures (data not shown). However, another Complex I inhibitor amobarbital and MPTP inhibitor CsA were effective and significantly prevented hypoxia-induced increase in LDH release (Figure 6b). These compounds had no effect on the level of necrosis under normoxic conditions (data not shown).

The data show that effects of rotenone, amobarbital and CsA on simulated ischaemia- or hypoxia-triggered cell death in brain slice cultures is different among age groups investigated. All inhibitors efficiently prevented ischemia-induced necrosis in 5–7 days brains, and amobarbital and CsA (but not rotenone)—hypoxia-induced necrosis in adult rat brains.

### 3.4. Acute Effects of Inhibitors of Complex I and MPTP on Ca^2+^ Retention Capacity of Brain Cortex Mitochondria

In the next series of experiments we investigated whether age of rats may cause differences on Ca^2+^- induced MPTP regulation in isolated brain mitochondria. The respiratory rates (with pyruvate plus malate as substrates) of mitochondria isolated from premature rat brains were: LEAK respiration (State 2) −98 ± 15 pmolO_2_/s/mg protein and OXPHOS (ADP-stimulated respiration) −383 ± 33 pmolO_2_/s/mg protein; for mitochondria isolated from adult rat brains—LEAK −175 ± 8 pmolO_2_/s/mg protein and OXPHOS −788 ± 90 pmolO_2_/s/mg protein. These data indicate high quality of mitochondrial preparations of both age groups.

**Figure 7:**
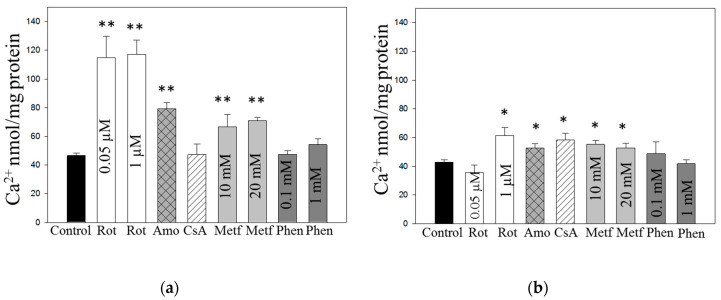
Ca^2+^ retention capacity of brain cortex mitochondria isolated from 5–7 days (**a**) and 2–3 months (**b**) old rats. Mitochondrial calcium retention capacity (CRC) was measured fluorometrically using Calcium Green-5N as described in Methods. Where indicated, rotenone (Rot, 0.05 and 1 µM), amobarbital (Amo, 2.5 mM), cyclopsorine A (CsA, 0.5 µM), metformin (Metf, 10 and 20 mM) or phenformin (Phen, 0.1 and 1 mM) were added to isolated mitochondria 5 min before calcium pulses. The data are presented as means ± standard errors of 3–10 separate experiments. ∗—*p* < 0.05, ∗∗—*p* < 0.01 compared to the control of corresponding age group.

MPTP in the experiments was evaluated as mitochondrial calcium retention capacity (CRC). There was no significant difference in CRC of isolated brain mitochondria comparing 5–7 days and 2–3 month age groups (Figure 7a). CRC of 5–7 days old rat brain was insensitive to cyclosporine A, though it was significantly higher in the presence of Complex I inhibitors: CRC was increased by about 150% with rotenone, by 70% with amobarbital and by about 50% with metformin, compared to control (Figure 6a). Similarly, in 2–3 months age group, amobarbital increased CRC by 23% and metformin by 23–29%, however only higher −1 µM rotenone concentration increased CRC by 43% (Figure 7b). Adult rat brain mitochondria were sensitive to cyclosporine A which increased CRC by 38%. However, phenformin had no effect on brain mitochondrial CRC in both, 5–7 days and 2–3 months, age groups. Altogether, the data suggest that MPTP in premature rat brains is more sensitive to acute effects of Complex I inhibitors than mitochondria from adult brains whereas cyclosporine A acutely desensitized MPTP to calcium in adult brain mitochondria.

### 3.5. Effects of Metformin, Phenformin, and Inhibitors of Complex I on MPTP Opening in Intact Cultured CGC Neurons

**Figure 8:**
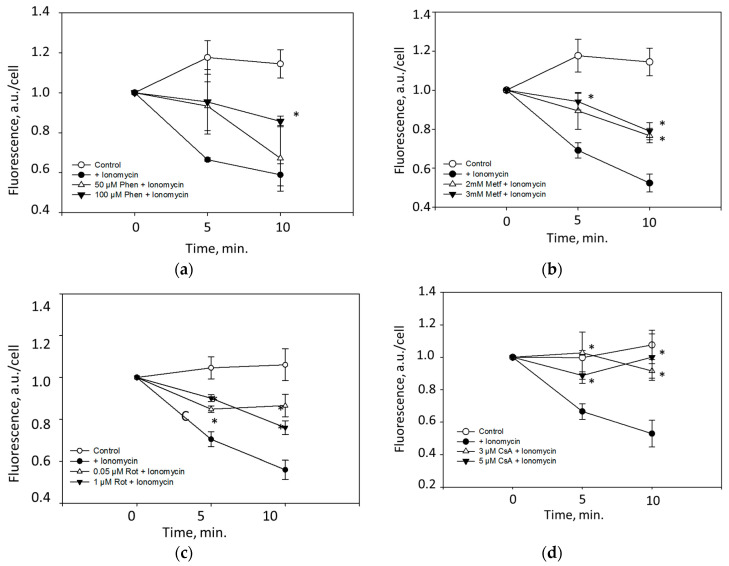
Effects of phenformin, metformin, rotenone and cyclosporin A on MPTP opening in neuronal cells. Pure neuronal cultures prepared treating CGC cultures with Ara-C (see Section 2.2) were pre-incubated for 1 h with: (**a**) 50 μM and 100 μM phenformin (Phen), (**b**) 2 mM and 3 mM metformin (Met); (**c**) 0.05 μM and 1 μM rotenone (Rot); and (**d**) 3 μM and 5 μM cyclosporine A (CsA). Mitochondrial permeability transition pore (MPTP) opening was quantified by measuring fluorescence alteration after staining with Calcein-AM as described in Methods. The data were normalized against controls of each group assuming that control level is 100% and presented as mean ± standard errors of 4–6 experiments on separate cell cultures. ∗—*p* < 0.01.

We tested whether metformin and phenformin exert an effect on MPTP in intact neuronal cells. In these experiments, pure neuronal cultures were pre-incubated 30 min with phenformin or metformin and then cells were loaded with fluorescent agent Calcein-AM which enters into cells and accumulates in mitochondria. Calcein fluorescence in the cytosol is then quenched with CoCl_2_. MPTP opening was induced in these cells by adding selective calcium ionofore—ionomycin. As shown in Figure 8, after addition of ionomycin there was a rapid decrease in fluorescence which is an indicator of MPTP opening allowing efflux of fluorescent calcein from mitochondria into cytosol. However, in neurons pre-incubated with 100 µM phenformin the decrease in fluorescence was substantially lower over 10 min incubation period (Figure 8a). Lower concentration of phenformin was less effective. Similarly, metformin at 2 mM and 3 mM concentrations substantially reduced ionomycin-induced decrease in fluorescence suggesting prevention of MPTP opening (Figure 8b). Selective inhibitor of Complex I—rotenone effectively reduced ionomycin-induced MPTP opening even at lowest 0.05 µM concentration (Figure 8c). Ionomycin-induced decrease in fluorescence in neurons was also suppressed by cyclosporine A—a specific inhibitor of MPTP (Figure 8d). Altogether, these data suggest that phenformin and metformin can suppress MPTP in neurons possibly acting via Complex I inhibition.

## 4. Discussion

The study was designed to compare the effects of hypoxia and simulated ischemia (hypoxia plus DOG) on the brain slices cultures prepared from premature (5–7 days) and adult (2–3 months old) animals and to test whether two biguanide derivatives—metformin and phenformin—exert protective effects against hypoxic/ischemic damage and by which mechanism. We found that premature brain slice cultures were resistant to 24 h hypoxia alone; however, hypoxia in the presence of DOG (simulated ischemia) induced necrosis in these brain slice cultures. Treatment of premature brain slice cultures with phenformin before simulated ischemia protected from necrotic cell death whereas pretreatment with metformin was practically not effective against ischemia-induced necrosis in these brain slice cultures.

Both compounds, metformin and phenformin, at concentrations that were protective against cell death, caused direct partial inhibition of Complex I activity in isolated brain mitochondria. The molecular structure of phenformin slightly differs from metformin: Compared to metformin, phenformin has additional phenyl ring and a two-carbon linker between the phenyl ring and the biguanide moiety [42]. According to the literature, these structural features may lead to a significantly stronger inhibition of Complex I due to hydrophobic properties and easier transport to the mitochondrial matrix [19]. The data presented in this study are in agreement with this as we showed that about 10–20 times lower concentration of phenformin was needed to inhibit Complex I activity to the similar level as metformin.

Interesting and unexpected finding of this study was that adult rat brain slices (2–3 months old) were sensitive to hypoxia alone: In this case 24 h hypoxia induced substantial necrosis which was prevented in the presence of DOG. The results with cultures of 5–7 days old rat brain slices were opposite—DOG supplement caused greater ischemic necrotic damage, whereas in the absence of DOG there were no differences between normoxic and hypoxic levels of necrosis. These data correlate with findings by other researchers showing that 5–7 days old rats brain slice cultures were more sensitive to decreased glucose than slices from 2–3 months and older rats [43]. The exact mechanism of how DOG reduces hypoxia-induced cell death in adult rat brains is not clear, but inhibition of glycolysis by DOG may involve activation of some pro-survival signaling pathways, like hypoxia-inducible factor-1α [44], signal transducer and activator of transcription 3 and transcription factor 4 [45,46].

Hypoxia-induced necrosis in slice cultures from adult brains was found to be prevented by pre-treatment with both phenformin and metformin. These findings are in agreement with previously reported studies by other investigators: Zeng and colleges performed study in vivo with 2–3 month old mice and showed that metformin administered intraperitoneally immediately after induction of cerebral ischemia increased neuronal survival [46]. In another study with 2–3 month old rats administration of metformin by 1, 3, or 7 days before stroke resulted in the reduction of cerebral infarction volume [47]. Absence of the effect of metformin in 5–7 days age group observed in our study is not entirely clear, however it can be explained by the difference of energy metabolism that occur with development and aging. For example, in the study of healthy human population with age between 5 and 106 years, there was observed a shift in the glucose metabolism from aerobic to anaerobic, influencing the oxygen consumption not completely coupled with the ATP synthesis and increase in ROS production [48].

Importantly, in this study we demonstrated that necrosis induced by simulated ischemia in premature brain slice cultures as well as hypoxia-induced in adult brain slice cultures was prevented by a specific inhibitor of MPTP—cyclosporine A, suggesting that this cell death was mediated by MPTP. Necrosis in premature brain slices was also prevented by rotenone—a selective inhibitor of mitochondrial Complex I as well as by amobarbital which also inhibits Complex I. Amobarbital also prevented hypoxia-induced necrosis in adult brain slice cultures though rotenone was less effective in this case. We showed that preincubation of neuronal cell cultures with rotenone, phenformin and metformin substantially prevented cyclosporine-A-sensitive ionomycin-induced MPTP opening in intact neuronal cells suggesting that MPTP in neurons may be regulated by Complex I activity and that phenformin and metformin can inhibit MPTP. This allows to suggest that protective effects of phenformin in premature and adult brain slice cultures as well as effect of metformin in adult brain slice cultures against ischemia/hypoxia-induced cell death may be related to inhibition of MPTP due to suppression of Complex I activity. This is in line with our previous study, where we showed that infusion of rotenone to adult rats protected from brain ischemia-induced cell death by inhibiting MPTP [49], as well as data of other researchers who demonstrated that inhibitors of Complex I amobarbital [9,50,51] and rotenone [8] decreased dysfunction of myocardial mitochondria and ischemic heart injury.

Further support for our conclusion was provided by assessment of calcium retention capacity of isolated brain mitochondria which was found to be increased by rotenone, amobarbital and metformin. The results of these experiments also revealed age-dependent differences in modulation of MPTP by Complex I inhibitors and cyclosporine A which inhibits MPTP by binding to cyclophilin D. MPTP in brain mitochondria isolated from premature (5–7 days old) rats were more sensitive to Complex I inhibitors—rotenone, amobarbital and metformin than in adult rat brain mitochondria where higher concentrations of rotenone were required to delay MPTP opening, as well as lower inhibition of MPTP by amobarbital and metformin was observed. Meanwhile MPTP in mitochondria from adult rat brains were more sensitive to cyclosporine A whereas MPTP in premature brain mitochondria were not acutely affected by the same concentration of cyclosporine A. Note that in neurons isolated from 7-day-old rats, cyclosporine A suppressed MPTP opening but at higher concentrations than used for isolated mitochondria indicating that even at that age neuronal MPTP can be modulated by cyclophilin D inhibitor.

It is important to mention that concentrations of metformin that inhibited MPTP in isolated brain mitochondria were higher than used in cultured neurons and exhibited protective effects in brain slice cultures. Phenformin even at the highest 1 mM concentration had no direct acute effect on CRC of isolated brain mitochondria though it inhibited ionomycin-induced MPTP opening in intact neurons at much lower concentration. One of the possible explanations for such findings may be related to different affinity of biguanides to Complex I conformational states [19,52]. Complex I can exist in active or deactive conformation, and ischemia has been shown to promote deactivation of this complex [52]. In the heart, metformin has been shown to inhibit Complex I in ischemia-damaged but not control mitochondria [10]. Our data show that Complex I is inhibited by metformin and phenformin in control mitochondria disrupted by freezing–thawing—a procedure which may affect conformational state of Complex I. Therefore it is possible to speculate that in isolated intact brain mitochondria Complex I is in active form which is less sensitive to biguanides. In intact cells (cultured neurons or brain slice cultures), Complex I may be in deactive conformation, particularly under hypoxic conditions, and this may promote phenformin and metformin binding and inhibiting Complex I. Recently, Matsuzaki and Humphries demonstrated supporting data that biguanides selectively inhibit the deactivated form of complex I [51]. Thus lower concentrations of biguanides would be needed to suppress MPTP in intact cells, particularly exposed to hypoxia. However, such hypothesis needs further experimental investigation.

We cannot completely exclude other possible mechanisms of protective action of metformin and phenformin. It has been previously suggested that metformin suppresses oxidative stress in rat brain after ischemia and ischemia/reoxygenation [53]. Other investigators have proposed that treatment with metformin increases cerebral AMPK activation following ischemia [54], suggesting that metformin may confer neuroprotection by activating AMPK [55,56]. Other authors obtained contradictory data indicating that AMPK is highly expressed in neurons and is rapidly activated in the brain during energy-deprived states such as ischemia, though metformin ameliorated stroke-induced activation of AMPK [56]. However, recent study by Lesnefsky and colleges (2019) with isolated cardiac mitochondria and cultured H9c2 cells affected by ischemia suggested that protective effect of metformin is independent of AMPK activation [10]. Their data support our findings in this study that treatment of metformin before ischemia protects cells from ischemia-induced injury by inhibiting activity of Complex I and improving mitochondrial calcium retention capacity and delaying MPTP opening.

The exact mechanism of regulation of MPTP opening by Complex I activity is unclear but may involve suppression of production of ROS (which are activators of MPTP) due to prevention of reverse electron transfer through the respiratory chain (a condition which may arise during ischemia due to accumulation of succinate) [10]. Another possible explanation is that Complex I activity may affect ubiquinone redox state, which in turn may modulate MPTP [15]. The third possibility is that Complex I may be involved in formation of MPTP, thus inhibitors binding to the complex may affect its structure depending on the redox state and subsequently modulate MPTP [16].

## 5. Conclusions

The current study suggests that mitochondrial Complex I is one of the important targets in ischemia-induced injury. Pharmacological compounds such as metformin and phenformin that act on its activity may regulate opening of MPTP and lead to survival of brain cells under ischemic conditions.
